# The Expression Levels of MicroRNA-361-5p and Its Target VEGFA Are Inversely Correlated in Human Cutaneous Squamous Cell Carcinoma

**DOI:** 10.1371/journal.pone.0049568

**Published:** 2012-11-14

**Authors:** Alexander Kanitz, Jochen Imig, Piotr J. Dziunycz, Adriana Primorac, Alessia Galgano, Günther F. L. Hofbauer, André P. Gerber, Michael Detmar

**Affiliations:** 1 Institute of Pharmaceutical Sciences, ETH Zurich, Zurich, Switzerland; 2 Department of Dermatology, University Hospital Zurich, Zurich, Switzerland; Ohio State University Medical Center, United States of America

## Abstract

Vascular endothelial growth factor A (VEGFA) plays a key role in the angiogenesis of human skin. Elevated levels of VEGFA are associated with several pathological conditions, including chronic inflammatory skin diseases and several types of skin cancer. In particular, squamous cell carcinoma (SCC) of the skin, the second most common skin cancer in the general population, is characterized by invasive growth, pronounced angiogenesis and elevated levels of VEGFA. The processing, turnover and production of VEGFA are extensively regulated at the post-transcriptional level, both by RNA-binding proteins and microRNAs (miRNAs). In the present study, we identified a new miRNA recognition element in a downstream conserved region of the VEGFA 3′-UTR. We confirmed the repressive effect of miR-361-5p on this element *in vitro*, identifying the first target for this miRNA. Importantly, we found that miR-361-5p levels are inversely correlated with VEGFA expression in SCC and in healthy skin, indicating that miR-361-5p could play a role in cancers.

## Introduction

Angiogenesis is a multi-step process leading to the formation of vascular structures derived from preexisting blood vessels, either through remodeling or the formation (“sprouting”) of new vessels. It involves the induction of microvascular hyperpermeability, breakdown of the vascular basement membrane, recruitment and proliferation of endothelial cells (EC), and the formation of mature blood vessels. Positive and negative regulators of angiogenesis have to be tightly regulated to maintain physiological tissue homeostasis and function. For example, in healthy adult skin, angiogenesis is generally quiescent as angiogenic stimuli are overruled by inhibitory signals. However, environmental insults may tip this balance, leading to initiation of angiogenesis in order to counteract tissue damage. Similarly, excessive angiogenesis in the skin, resulting from dysregulation of one or more of its regulators, is associated with a plethora of pathological conditions, such as psoriasis and other inflammatory dermatoses, autoimmune blistering diseases, and many cancers, most prominently melanoma, basal cell carcinoma and squamous cell carcinoma. Anti-angiogenic therapy therefore holds promise for the treatment of a wide spectrum of human ailments [1], [2].

Cutaneous squamous cell carcinoma (SCC) is the second most common skin cancer in the general population [3]. In contrast to basal cell carcinoma – the most common skin cancer – it is characterized by the risk for metastasis. Incidence of SCC is 60- to 100-times higher among immunosuppressed patients, which makes it the most common cancer following organ transplantation. Invasive SCC develops from atypical keratinocytes, clinically visible as actinic keratosis or Bowen’s disease, both considered intraepithelial non-invasive forms of SCC [4]. Approximately 1% of these intraepithelial lesions develop into an invasive SCC [5]. However, such tumor development requires intense interactions with stromal cells and profound extracellular remodeling. Angiogenesis is an essential part of the malignant phenotype as most tumors are apparently not able to exceed 1–2 mm of diameter without developing new blood vessels [6]. Therefore they produce angiogenic factors at an early point of development.

Vascular endothelial growth factor A (VEGFA) is a homodimeric heparin-binding glycoprotein that mainly acts as a paracrine mitogen, growth and survival factor for ECs, but it also causes vascular permeability, vasodilatation, and various changes in immune cell properties upon binding to its main receptors VEGF receptor −1 and −2. VEGFA has been identified as the predominant tumor angiogenesis factor in the majority of human cancers, including those of the breast, colon, lung and prostate [7], [8]. Invasive SCC also expresses increased levels of VEGFA, particularly in the leading front of the tumor, which is an intuitive site for the induction of angiogenesis [9]. Furthermore, overexpression of (murine) VEGFA resulted in enhanced invasion and angiogenesis in experimental human SCC [10]. In some other cancers, such as head and neck squamous cell carcinoma, increased expression of VEGF has been associated with progression to a more aggressive phenotype, both clinically and in experimental systems [11]. Similarly, increased VEGFA expression correlates with greater metastatic potential of melanoma, and its expression is high in melanoma metastases themselves [12], [13]. In the skin, VEGFA is mainly secreted by epidermal keratinocytes. Its expression is up-regulated in response to hypoxia [14], activation of epidermal growth factor (EGF) receptor signaling via EGF or transforming growth factor (TGF)-α, and to a number of cytokines including tumor necrosis factor (TNF)-α, TGF-β, fibroblast growth factor-7 and others [15], [16].

Interestingly, it was shown that heterozygous deletion of the VEGFA 3′-untranslated region (3′-UTR) in mice leads to a two- to three-fold increase in VEGFA levels and embryonic lethality following cardiac failure, thus suggesting the presence of important regulatory elements in its downstream untranslated region [17]. Indeed, VEGFA expression appears to be excessively regulated at the post-transcriptional level. For example, at least twelve protein isoforms have been described, resulting from alternative splicing, as well as the use of alternative promoters or ribosome entry sites [18]. Moreover, its 3′-UTR contains several putative or verified stabilization/destabilization elements and alternative polyadenylation signals, which may influence mRNA processing, turnover and translation rates upon binding of the respective trans-acting factors, such as ELAV1/HuR, PTB or the γ-interferon activated inhibitor of translation (GAIT) complex [19]–[34].

Various microRNAs (miRNAs) have been found to regulate human VEGFA expression post-transcriptionally. MiRNAs are short (∼21 nucleotides) noncoding RNAs that generally lead to repression of gene expression upon the binding of recognition motifs that are typically present in the 3′-UTR of transcripts. Binding occurs in an antisense fashion which is mainly mediated by a ‘seed’ region which comprises the 5′-terminal six to eight nucleotides of the miRNA and exhibits a high degree of complementarity to the target mRNA. Misexpression of miRNAs in hypoxia-induced nasopharyngeal carcinoma-derived CNE cells identified several miRNAs that were able to repress VEGFA expression *in vitro*, although the corresponding recognition motifs in the mRNAs have not been unambiguously identified [35], [36]. One of the two VEGFA internal ribosome entry sites, driving the expression of the VEGF-121 isoform [37], has been shown to be susceptible to regulation by miR-16 [38]. Moreover, miR-16, as well as miR-424, have recently been demonstrated to regulate angiogenic activity in endothelial cells by targeting VEGFA and other angiogenic mediators [39]. Lei *et al.* identified a feedback loop regulating the adaptation of murine tumor cells to different oxygen concentrations in which hypoxia-inducible factor 1 alpha (HIF-1α) suppresses the expression of miR-20b, which in turn may regulate both HIF-1α and VEGFA expression [40]. Two miRNAs, miR-126 and -205, were shown to regulate VEGFA expression and are implicated in a number of cancers, including lung, prostate and breast cancer [41]–[46]. Similarly, miR-200c is dysregulated in leiomyomata [47] and endometrial cancers [48], while miR-29b-mediated regulation of VEGFA is implicated in prostate cancers [45]. Jafarifar *et al.* showed that the binding of the splicing factor hnRNP L to a CA-rich sequence element in the VEGFA 3′-UTR under hypoxia led to competitive displacement of miRNAs miR-297, -299, -567 and -605 and consequently derepression of VEGFA expression in tumor-associated macrophages [30]. Finally, microRNAs 200b, 93 and 29b were implicated in the non-malignant conditions diabetic retinopathy [49], diabetes [50], and pre-eclampsia [51], respectively.

In the present study, we have used miRNA target prediction algorithms and luciferase reporter assays to identify a new microRNA recognition element (MRE) in a downstream conserved region of the VEGFA 3′-UTR, and we have confirmed the repressive effect of miR-361-5p on VEGFA expression *in vitro* with luciferase reporter assays and ELISA. We also found that miR-361-5p levels were lower in those skin-derived cell lines that express high levels of VEGFA, as well as in SCC tumors compared to healthy skin. These results indicate that miR-361-5p might affect cancer development or progression by modulating VEGFA expression in particular tumor types.

## Results

### VEGFA is a Putative Target of microRNA-361-5p

The almost 2 kb long sequence of the human VEGFA 3′-UTR, the vast majority (>95%) of which is present in all of its known isoforms, contains two regions that are highly conserved among vertebrates, one at its 5′- and the other one at its 3′-end. While most miRNA recognition elements (MREs) that have been unambiguously shown to be able to affect human or murine VEGFA expression are located in the 5′-conserved region [30], [38]–[40], [42], [50], only miR-126, miR-200b/c, and recently miR-29b have been demonstrated to bind in the ∼730 nucleotide downstream conserved region (Figure S1A) [41], [43]–[45], [47]–[49], [51], [52]. Both regions are lower in GC content compared to the weakly conserved region separating them (GC% approximately 44, 58 and 28 from 5′ to 3′). It has also been suggested that the density of functional MREs increases towards both ends of a transcript’s 3′-UTR [53]. We therefore wondered whether the 3′-conserved region of the VEGFA 3′-UTR contains additional MREs that may contribute to VEGFA dysregulation in cancers.

In order to find potential candidate miRNAs that may be implicated in the regulation of VEGFA expression, we employed five miRNA target prediction services to search for predicted MREs in the VEGFA 3′-UTR: microRNA.org [54], TargetScan [55], DIANA-microT [56], miRDB [57], and MicroCosm [58]. This analysis revealed that only three miRNA/MRE pairs were predicted by all five algorithms (Table S1): Two MREs, predicted to be targeted by miR-29b and miR-205, have already been shown to regulate VEGFA expression [42], [45], [46], [51]; the third MRE, predicted to be regulated by miR-361-5p, is located in the downstream conserved region. RNAhybrid [59] calculated a minimum free energy of −22.0 kcal/mol for the interaction between miR-361-5p and the MRE located between nucleotides 1604 and 1625 of the VEGFA 3′-UTR in transcript NM_001025366 (Figure S1B), which is in the range of other MREs reported for VEGFA [36]. No targets for miR-361-5p have been experimentally confirmed so far, but it has been shown that the transfection of a miR-361-5p mimic in hypoxia-induced CNE cells leads to reduced VEGFA protein levels, as determined by ELISA – supporting the idea that this miRNA may regulate VEGFA expression [35], [36]. Thus, we chose to focus our studies on this miRNA.

*MIR361* is encoded on the × chromosome, in an intron between exons 9 and 10 of *CHM*/choroideremia (Rab escort protein 1) and gives rise to two mature miRNA species, miRNA-361-3p and the predominant miRNA-361-5p. The locus is highly conserved among placental mammals only, particularly the stem region of the putative precursor miRNA. MicroRNA 361-5p has first been isolated from pancreatic islets by Poy and colleagues [60] and subsequently from neuroblastoma cell lines [61]. In the following, the miRNA has been detected in different tissues and cell types [62]–[67]. Moreover, in mice the miRNA may be involved in the molecular mechanism underlying insulin resistance and protection thereof [68], and it was found to be differentially expressed in a bleomycin-induced mouse model of lung fibrosis [69].

It is of interest that miR-361-5p is predicted to target mRNAs coding for other proteins acting in angiogenesis (a list of potential miR-361-5p targets compiled from five miRNA target prediction algorithms is given in Table S2; pathways enriched among the Gene Ontology annotations of candidates are shown in Table S3; putative targets acting downstream of VEGFA are highlighted in a pathway map in Figure S2). Potential downstream targets include PKC, Rac, PI3K, NFAT. The pathways most strongly enriched among predicted miR-361-5p targets include upstream regulators of VEGFA expression, such as the EGF and FGF signaling pathways (P = 7.2 × 10^−6^ and 2.6 × 10^−5^, respectively), as well as the VEGF pathway and angiogenesis (P = 0.0085 and 0.0016).

### MicroRNA-361-5p Targets the VEGFA 3′-UTR

**Figure 1:**
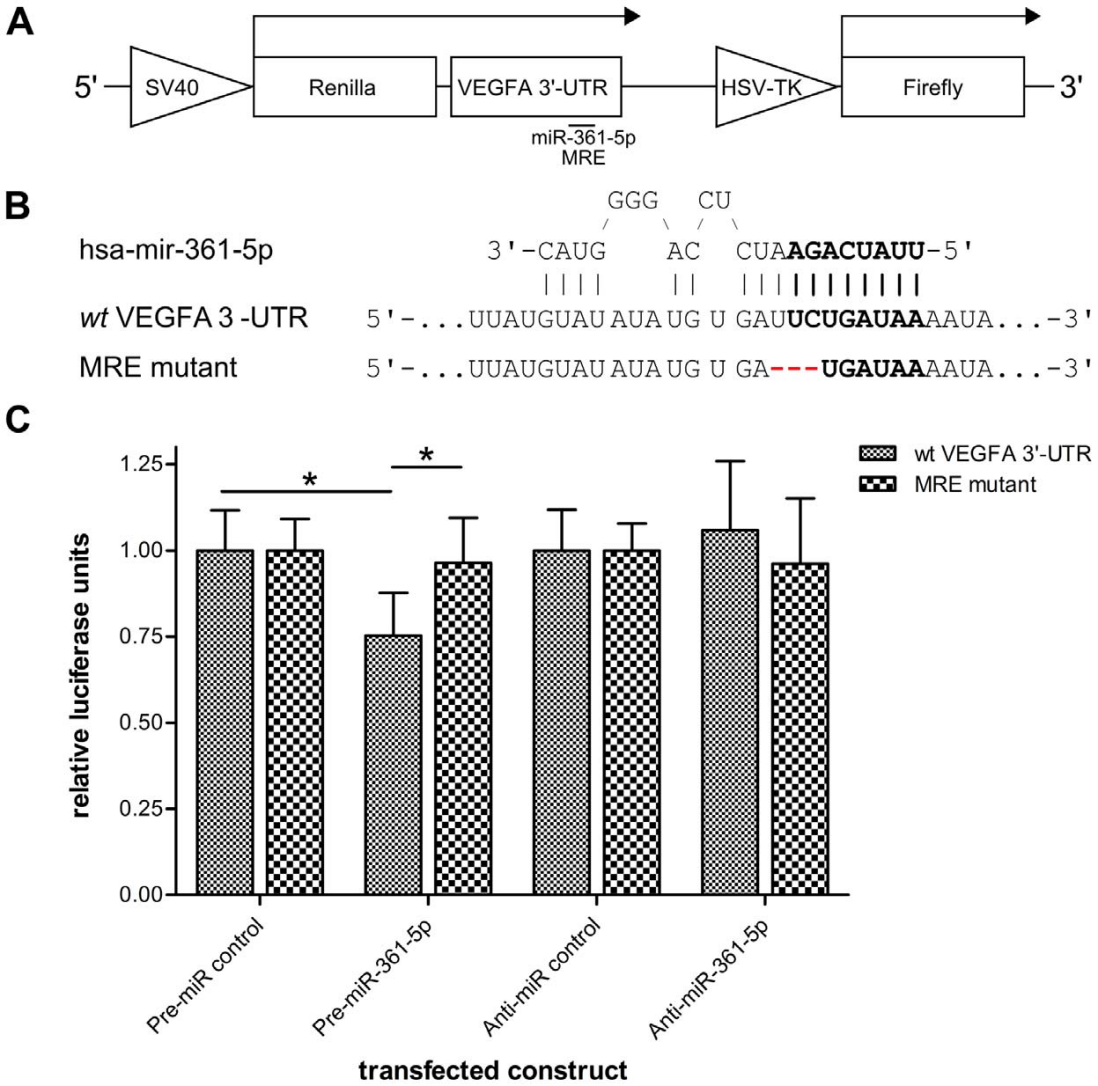
Mutation of the putative recognition element abolishes miRNA-361-5p-mediated regulation of a VEGFA 3′-UTR reporter. (A) Schematic representation of the luciferase reporter constructs, indicating the VEGFA 3′-UTR fragment fused to *Renilla* luciferase, the predicted miRNA recognition element (MRE) for miR-361-5p, and the firefly luciferase gene used for normalization. (B) Sequence alignment of miR-361-5p with the wild type (wt) VEGFA 3′-UTR and mutated reporter. The seed sequence (bold) and mutated residues (red) are highlighted. (C) HEK293 cells were co-transfected with either of the indicated luciferase reporters and a miR-361-5p mimic, antisense inhibitor, or the respective control. Data were obtained from three independent experiments, performed in triplicate. For each experiment, mean ratios of *Renilla* and firefly luciferase activities were normalized to those of psiCHECK-2-transfected cells, then to Pre- or Anti-miR control-transfected cells. Mean values of three independent experiments ± S.D. are plotted. Two-tailed, unpaired *t*-tests were used to calculate P values (single asterisks denote P values<0.05).

Due to the generally high degree of interconnectivity within post-transcriptional regulatory networks [70]–[73], the extensive post-transcriptional regulation that has already been shown to be exerted on the VEGFA transcript [19]–[52], and the high occurrence of predicted MREs in the conserved region surrounding the putative MRE (Table S1), it is possible that other *trans*-binding factors might affect the binding potential of our miRNA of interest. Thus we reasoned that it may be beneficial to preserve potential RNA recognition elements in our experiments. To do this, we cloned the entire downstream conserved region of the VEGFA 3′-UTR behind the coding sequence of *Renilla* luciferase under the control of an SV40 promoter, on a plasmid further encoding a firefly luciferase for normalization purposes (Figure 1A). Additionally, we also generated a mutant of the putative miR-361-5p MRE, in which three nucleotide residues are deleted (Figure 1B; effective deletion relative to the miRNA seed region = 2 nucleotides). In order to avoid competition between the reporter and endogenous VEGFA, we performed the assays in human embryonic kidney (HEK293) cells [74], which express low levels of VEGFA [75].

The ability of HEK293 cells to take up miRNA mimics (see materials and methods) was verified by transfection with increasing amounts of a Cy3-labeled control oligonucleotides, followed by flow cytometry analysis (Figure S3), revealing that>90% of the cells take up the labeled mimics across the whole range of concentrations in a dose-respondent fashion. When overexpressing the miR-361-5p mimic together with the wild type VEGFA 3′-UTR luciferase reporter, relative *Renilla* activity was decreased by approximately 25% compared to the control precursor (P = 0.0327; unpaired *t*-test, two-tailed), as well as the mutated reporter (P = 0.0263; unpaired *t*-test, two-tailed), which did not change relative to the control (Figure 1C). Although the co-transfection of wild type and mutant reporters with miR-361-5p antisense inhibitor or control did not lead to significant differential luciferase activities, these data indicate that the putative miR-361-5p MRE possesses regulatory potential, and that it is subject to regulation by miR-361-5p.

### Endogenous VEGFA Expression is Regulated by microRNA-361-5p

**Figure 2:**
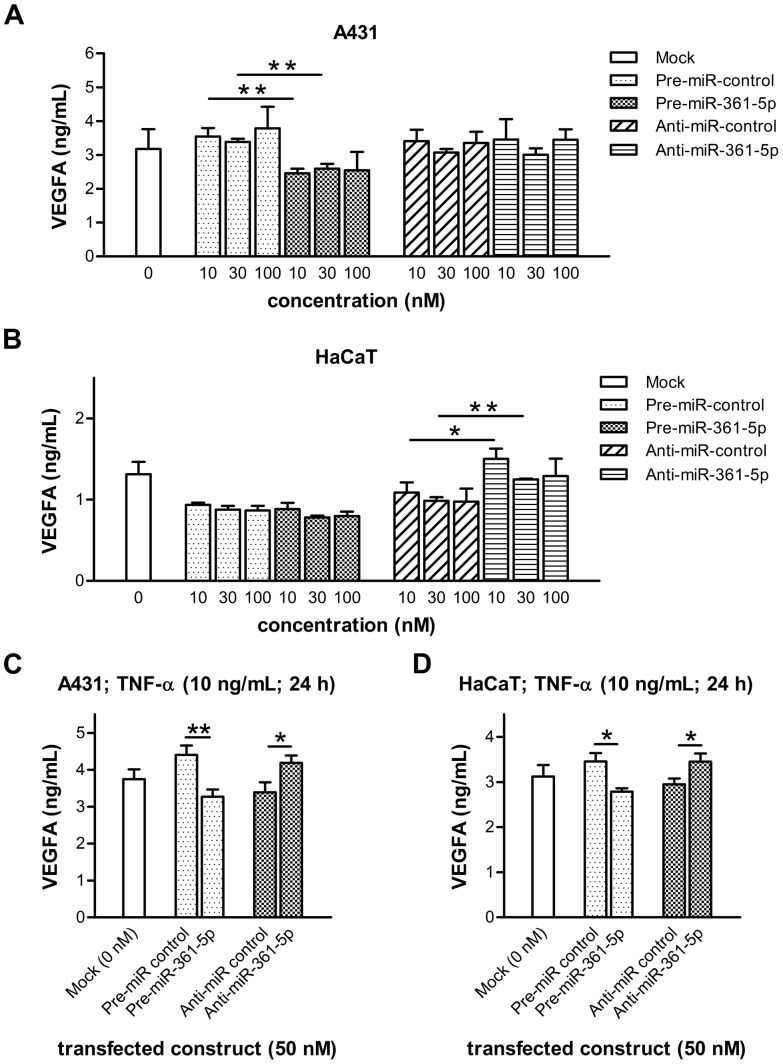
Impact of altered miRNA-361-5p levels on secretion of endogenous VEGFA. A431 and HaCaT cells were transfected with the indicated concentrations of miR-361-5p mimics, antisense inhibitors, or controls, either in the presence (A, B) or absence (C, D) of TNF-α. Cellular supernatants were analyzed for VEGFA protein content using a human VEGFA ELISA. Three independent experiments were performed in triplicate. Mean values ± S.D. from a representative experiment are plotted. Two-tailed, unpaired *t*-tests were used to calculate P values (one and two asterisks denote P values<0.05 and<0.01, respectively).

To check whether endogenous VEGFA levels could be affected by miR-361-5p, we chose to alter levels of the miRNA in epidermoid squamous cell carcinoma-derived A431 cells [76] and keratinocytes derived from normal skin that transformed spontaneously *in vitro* (HaCaT) [77] – both of which are known to express high levels of VEGFA, as well as low levels of miR-361-5p (see the following paragraph). First, the efficiencies of transfecting these cells with miRNA mimics and antisense inhibitors were tested with increasing concentrations of Cy3-labeled control oligonucleotides and monitored by flow cytometry (Figure S3). Oligonucleotides were incorporated by more than 90% of the cells in all conditions, with fluorescence intensities increasing in a dose-respondent manner. We then determined VEGFA levels in the culture supernatants of both cell lines transfected with different amounts of miRNA-361-5p mimic or control using ELISA (Figure 2A/B). Baseline levels of secreted VEGFA of mock-transfected cells were approximately 3176±585 and 1314±152 pg/mL per 24 hours, for A431 and HaCaT cells respectively. VEGFA levels were significantly decreased in A431 cells (up to ∼30% when transfecting 10 nM; P = 0.0063; unpaired *t*-test, two-tailed; Figure 2A), while we only observed a slight decrease in VEGFA levels in HaCaT cells (up to ∼11% when transfecting 30 nM; P = 0.0502; unpaired *t*-test, two-tailed; Figure 2B) when comparing transfection of miR-361-5p mimic and control. Conversely, VEGFA levels were not affected in A431 cells transfected with increasing amounts of miRNA-361-5p antisense inhibitor when compared to cells transfected with a control (Figure 2A), while in HaCaT cells we detected elevated VEGFA levels for all tested antisense inhibitor concentrations (up to ∼39% when transfecting 10 nM; P = 0.0150; unpaired *t*-test, two-tailed; Figure 2B). Next we tested the effects of miR-361-5p mimics and antisense inhibitors in a setting where VEGFA expression is induced by treatment with TNF-α (10 ng/mL, 24 h; Figure 2C/D) [16]. As expected, baseline levels of VEGFA after 24 hours were increased by 18% (A431, 3745±266 pg/mL) and 138% (HaCaT, 3123±254 pg/mL) when compared to uninduced states (compare above). Importantly, significant changes in secreted VEGFA levels were found for miR-361-5p mimics and antisense inhibitors in A431 (∼26/24% decrease/increase for mimics and antisense inhibitors, respectively; P = 0.0045 and 0.0181; unpaired *t*-test, two-tailed; Figure 2C) and HaCaT cells (∼19/17% decrease/increase for mimics and antisense inhibitors, respectively; P = 0.0150 and 0.0233; unpaired *t*-test, two-tailed; Figure 2D), when compared to the corresponding control oligonucleotides. Taken together, these findings demonstrate that miR-361-5p affects the levels of secreted VEGFA, further suggesting that the miRNA is able to repress endogenous VEGFA expression *in vitro*.

### VEGFA and miR-361-5p Expression Levels are Inversely Correlated in Skin-derived Cells *in vitro*

**Figure 3:**
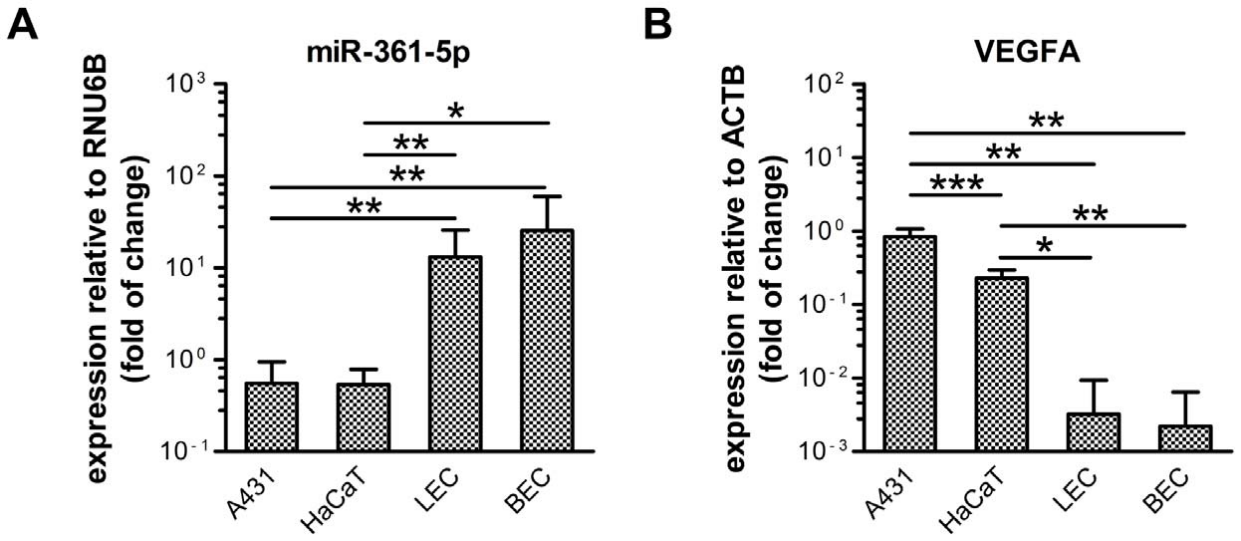
VEGFA and miR-361-5p expression levels are inversely correlated in human skin-derived cell lines. qRT-PCR analysis of miR-361-5p (A) and VEGFA (B) expression in A431, HaCaT, primary human dermal lymphatic (LEC) and blood endothelial cells (BEC). Fold differences ± S.D. in expression levels with regards to the references (RNU6B and ACTB, for miRNA-361-5p and miRNA-361-5p, respectively) are plotted. Data represent at least three independent experiments performed in duplicate. Two-tailed, unpaired *t*-tests were used to calculate P values (one, two and three asterisks denote P values<0.05,<0.01, and<0.001, respectively).

Next, we used quantitative reverse transcription PCR (qRT-PCR) to determine the expression levels of endogenous miR-361-5p and VEGFA mRNA in four skin-derived human cell lines: (1) the VEGFA-secreting epithelial cell lines A431 and HaCaT; (2) primary dermal lymphatic (LEC) and blood vascular (BEC) endothelial cells, which are targeted by VEGFA but express only low levels of it themselves [78], [79]. While miRNA expression did not differ significantly within the two groups of cells (fold difference between A431 and HaCaT = 1.04+0.97–0.50; fold difference between BEC and LEC = 1.94+3.77–1.28), miR-361-5p levels were significantly higher in the endothelial cells compared to the VEGFA-expressing epithelial cells (fold differences between LEC/BEC and A431/HaCaT ranging from 23.72 to 47.82; P values between 0.0064 and 0.0120; unpaired *t*-test, two tailed; Figure 3A). Conversely, while VEGFA mRNA levels differed only slightly within the two groups of cells (fold difference between A431 and HaCaT = 3.86+1.48–1.07; P = 4.0×10^−8^; fold difference between LEC and BEC = 1.46+5.12–1.13), the epithelial cells contained considerably higher amounts of VEGFA mRNA compared to the endothelial cells (fold differences between A431/HaCaT and LEC/BEC ranging from 70.43 to 378.41; P values between 0.0019 and 0.0101; unpaired *t*-test, two tailed; Figure 3B). With a Spearman’s rank correlation coefficient of the ΔC_T_ values of r = −0.80 (two-tailed) across all cell lines, the expression levels of miR-361-5p and VEGFA correlated well in an inverse manner, although this correlation did not reach significance. In summary, these data indicate that miR-361-5p is expressed in all of the tested skin-derived cell lines, with the highest levels occurring in endothelial cells. Considering the observed relation between miRNA and VEGFA levels, it is possible that miR-361-5p might play a role in maintaining the functional balance of differential VEGFA expression between epithelial and endothelial cells in the skin.

### MicroRNA-361-5p Levels are Decreased in Cutaneous Squamous Cell Carcinoma

Having established that miR-361-5p levels are lower in those skin-derived cell lines that exhibited higher levels of VEGFA expression *in vitro*, we hypothesized that its expression may be lower in SCC, and that it may thus contribute to the initiation or maintenance of high VEGFA expression, either directly or indirectly. We therefore measured the expression of miR-361-5p and several other VEGFA-regulating miRNAs in five samples of SCC obtained from patients and in five healthy skin samples using qRT-PCR.

**Figure 4:**
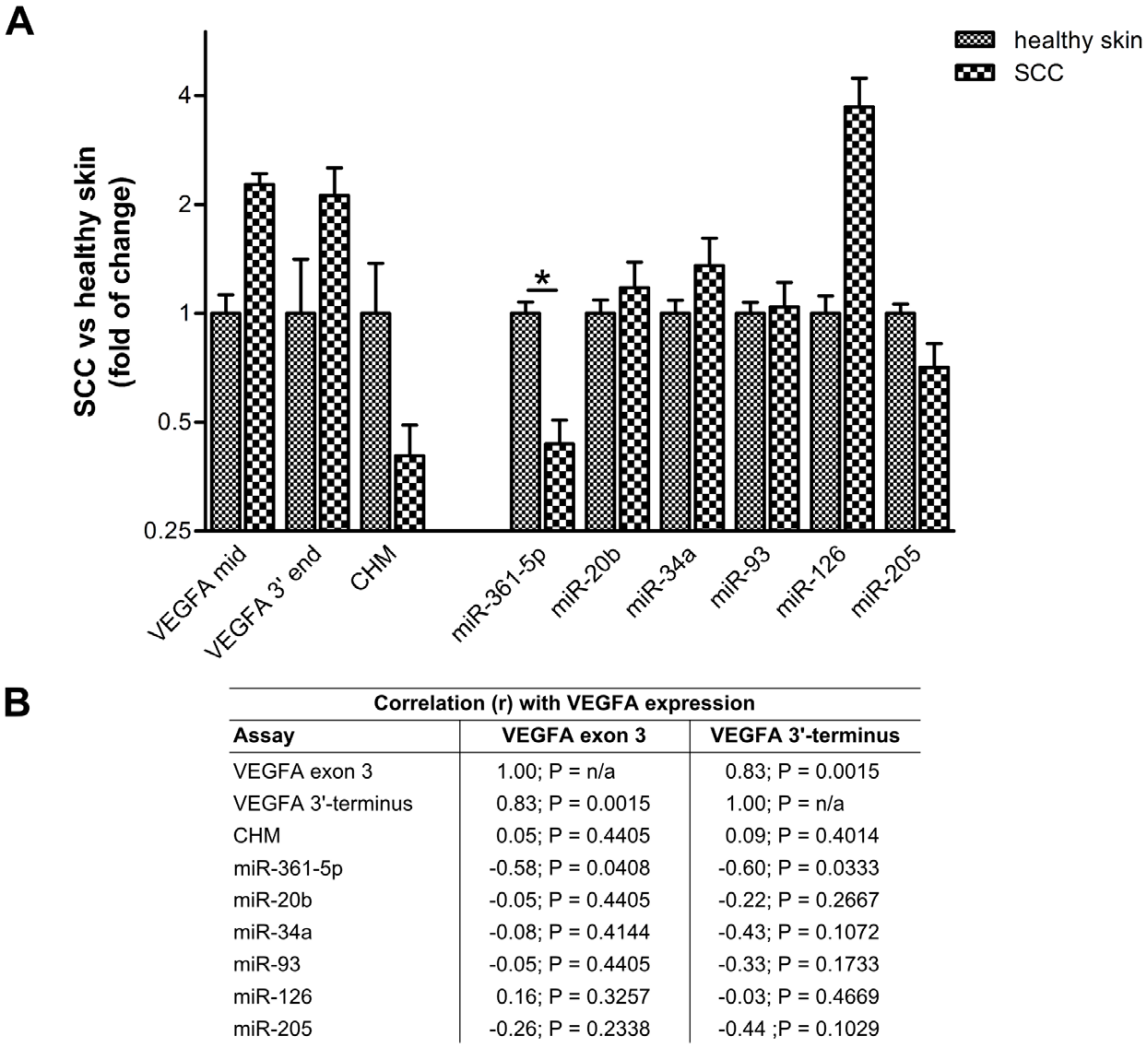
Relative changes in expression of selected mRNAs and mature miRNAs in healthy skin and SCCs. For each group, five samples were analyzed by qRT-PCR, representing ten individuals. Experiments were performed in quadruplicates. Data are based on C_T_ values normalized to ACTB and RNU6B for mRNAs and miRNAs, respectively. (A) Fold differences in expression relative to average of expression in healthy skin samples ± S.D. are indicated for each assay on a logarithmic scale. The Mann-Whitney *U* test was used to calculate P values (two-tailed; the single asterisk denotes a P value<0.05). (B) Based on the normalized expression levels, Spearman rank correlation coefficients (r) between relative expression levels of VEGFA and each of the indicated miRNAs were calculated over all samples. P values (one-tailed) are indicated.

First we investigated whether VEGFA expression was indeed increased in the SCC samples by assessing VEGFA mRNA levels with two different assays, one for exon 3 and the other one for the downstream conserved region in the 3′-UTR. Interestingly, we found that the VEGFA 3′-terminus was expressed at significantly lower levels than the coding region (fold difference between VEGFA exon 3 and VEGFA 3′-terminus assays of 2.72+1.34–0.90; P = 0.0098; unpaired *t*-test with Welch’s correction, two-tailed; P = 0.0115; Mann-Whitney *U* = 17, n1 = n2 = 10, two-tailed; Figure S4). As expected, we found that VEGFA mRNA levels were around two-fold higher in the SCC samples compared to healthy control samples (fold difference between SCC and healthy skin for the exon 3 assay: 2.27+2.61–1.22; P = −0.0472; unpaired *t*-test with Welch’s correction, two-tailed; P = 0.0556; Mann-Whitney *U* = 3, n1 = n2 = 5, two-tailed; fold difference between SCC and healthy skin for the 3′-terminal assay: 2.12+4.50–1.44; P = 0.1846; unpaired *t*-test with Welch’s correction, two-tailed; P = 0.2222; Mann-Whitney *U* = 6, n1 = n2 = 5, two-tailed; Figure 4A and S4B). Moreover, data correlated very well for both assays (r = 0.83, P = 0.0015; Spearman’s rank correlation, one-tailed; Figure 4B).

Next, we measured the expression of miR-361-5p and the known VEGFA-regulating miRNAs miR-20b, miR-34a, miR-93, miR-126 and miR-205. In order to gain first insights into the regulation of miR-361-5p, we also determined the expression levels of the miRNA’s ‘host gene’ *CHM*. In normal skin samples, the average expression levels of miR-20b (∼62-fold down) and miR-205 (∼51-fold up) strongly deviated from that of the reference RNA (RNU6B), while for all other miRNAs the difference stayed within an order of magnitude (Figure S4A). Of note, miR-361-5p levels (∼3.6-fold lower than RNU6B) were very consistent between samples (Figure S4A) and correlated fairly well with CHM mRNA levels (r = 0.53, P = 0.0587; Spearman’s rank correlation, one-tailed; data not shown). In the SCC samples, miR-361-5p and CHM levels were significantly decreased compared to healthy skin samples (fold difference between SCC and healthy skin for the miR-361-5p assay: 0.44+0.33–0.19; P = 0.0220; unpaired *t*-test with Welch’s correction, two-tailed; P = 0.0159; Mann-Whitney *U* = 1, n1 = n2 = 5, two-tailed; fold difference between SCC and healthy skin for the CHM assay: 0.40+0.53–0.23; P = 0.0456; unpaired *t*-test with Welch’s correction, two-tailed; P = 0.0952; Mann-Whitney *U* = 4, n1 = n2 = 5, two-tailed), whereas all other tested miRNAs either did not exhibit considerably reduced expression levels or, as seen for miR-126, even appeared to be increasingly expressed (fold difference between SCC and healthy skin of 3.72+10.99–2.78; P = 0.0699; unpaired *t*-test with Welch’s correction, two-tailed; P = 0.0952; Mann-Whitney *U* = 4, n1 = n2 = 5, two-tailed; Figure 4A and S4B). Importantly, miR-361-5p levels exhibited the strongest inverse correlation with VEGFA levels across all samples (r = -0.58 and -0.60, P = 0.0408 and 0.0333 for the exon 3 and 3′-terminal assays, respectively; Spearman’s rank correlation, one-tailed; Figure 4B). No other miRNA passed an r ≤−0.5 threshold. Notably, CHM did not correlate with VEGFA expression (r = 0.05 and 0.09, P = 0.4405 and 0.4014; Spearman’s rank correlation, one-tailed; for the exon 3 and 3′-terminal assays, respectively).

In summary, we found that out of a panel of six miRNAs targeting VEGFA, only miR-361-5p levels were decreased in SCC expressing high levels of VEGFA, indicating that miR-361-5p dysregulation could contribute to the observed elevated VEGFA levels in SCC. Although we did not observe a similar inverse correlation between CHM and VEGFA mRNA levels, the correlation between CHM and miR-361-5p levels may be an indication that transcription of the miRNA precursor is dependent on *CHM* transcription. Nevertheless, additional regulation at the miRNA processing step may occur and contribute to the non-correlated response of CHM and VEGFA transcripts [80].

## Discussion

In this study, we have identified microRNA 361-5p as a novel potential regulator of VEGFA expression. Using luciferase reporter assays, we have shown that miR-361-5p can target the VEGFA message via a recognition motif that is located in the downstream conserved region of the VEGFA 3′-UTR. Of note, an additional potential miR-361-5p MRE (located between nucleotides 1900 and 1921 of the 3′-UTR) was predicted by microRNA.org, and could contribute to the miRNA’s observed repressive potential, but was not analyzed by mutational analysis as part of this study because of the low prediction coverage and weaker calculated free minimum energy (-12.8 kcal/mol; RNAhybrid). Consistent with findings indicating that the regulatory impact of altered miRNA levels on endogenous protein levels of targets is often weak [81], [82], we have also observed mild inhibitory effects of miRNA 361-5p on the levels of secreted VEGFA. A difference in VEGFA miR-361-5p MRE occupancy by endogenous miR-361-5p between HaCaT and A431 cells could potentially explain the differential effects of miR-361-5p mimic or antisense inhibitor on the levels of secreted VEGFA in unstimulated cells: A high occupancy (‘saturation’) of the MRE in HaCaT cells, which express similar miR-361-5p but lower VEGFA levels compared to A431, could account for the absence of a significant decrease in VEGFA secretion upon the addition of miR-361-5p mimic. Consistently, stimulated HaCaT cells, which secreted>2-fold higher levels of VEGFA compared to unstimulated cells, responded to elevated levels of miR-361-5p. Conversely, a low occupancy of the MRE in unstimulated A431 cells could render the cells unresponsive to miR-361-5p inhibition. Likewise, the absence of an effect of miR-361-5p inhibition on luciferase reporter activity in HEK293 cells could also be explained by a relatively low MRE occupancy. However, data obtained from stimulated A431 cells, which express slightly more VEGFA than HaCaT cells, yet proved responsive to miR-361-5p inhibition, speaks against such a simple model. Moreover, in this scenario the impact of increased miR-361-5p levels in unstimulated A431 and miR-361-5p inhibition in unstimulated HaCaT cells should be proportional to the amount of added miRNA mimic or antisense inhibitor. The absence of such a miRNA dose-response could indicate that one or more additional factors differentially modulate miR-361-5p activity in the two cell lines, systemically or specifically, by influencing the availability, accessibility or functionality of the miRNA or its recognition element. The large set of known post-transcriptional regulators of VEGFA expression [19]–[52], as well as the extremely high density of predicted MREs in the downstream conserved region of the VEGFA 3′-UTR (0.41 MREs/nucleotide, compared to 0.29 and 0.14 for the upstream conserved and non-conserved regions, respectively, Table S1), add particular weight to such a hypothesis, as it implies the potential for a high degree of competition or similar cooperative effects between miRNAs, or between miRNAs and RNA-binding proteins. Indeed, such cooperation has already been observed for other miRNAs regulating VEGF expression *in vitro* [35]. Various studies have also demonstrated functional interplay between miRNAs and RNA-binding proteins on a number of targets [72], including VEGFA [30], and it has been proposed that such crosstalk may be widespread [83].

Furthermore, the localization of the MRE so close to the mRNA’s 3′-terminus may render it particularly prone to inaccessibility due to degradation or alternative polyadenylation. Regarding the latter, it has recently been proposed that proliferating cells may employ alternative polyadenylation to shorten the 3′-UTRs of transcripts and thus escape miRNA- and RNA-binding protein-mediated post-transcriptional repression [84]. Indeed, it was found that the VEGFA transcript uses two different polyadenylation sites in mice, although no differential usage of the signals was observed between normoxic and hypoxic conditions [85]. Similarly, our data indicate an apparent difference in expression levels between the coding region and the downstream region of the 3′-UTR within but not in between both clinical sample groups, suggesting that alternative polyadenylation may indeed limit the availability of the miR-361-5p MRE (Figure S4). However, slight differences in the amplification efficiencies of the used gene expression assays may also account for these observations. Further studies into the differential regulation of VEGFA 3′-UTR length, the interplay between miRNAs, as well as the crosstalk with other post-transcriptional regulators may shed light on these issues.

It cannot be ruled out that the ELISA results are due to secondary effects on non-targets or other miR-361-5p targets acting in the same pathway, which could in turn lead to altered VEGFA production/secretion. Indeed, while the reliability of miRNA target prediction software is often questionable at the level of individual targets, the strong enrichment of VEGFA-related pathways among predicted miR-361-5p targets (Table S2, Table S3, Figure S2) indicates that the miRNA may have potential roles in regulating angiogenesis and other VEGFA-related functions, both upstream and downstream of the VEGFA/VEGF receptor axis.

We have shown that miR-361-5p is expressed in a number of cell types derived from human skin. Interestingly, the epithelial, VEGFA-secreting cells (A431 and HaCaT) express significantly lower levels of this miRNA than the endothelial cells “targeted” by VEGFA (LEC and BEC). This may indicate a potential role for miR-361-5p in upholding the physiological balance of VEGFA levels in the skin. Furthermore, we have shown that levels of miR-361-5p, but not those of the known VEGFA-regulating miRNAs miR-20b, -34a, -93, -126, and -205, inversely correlate with VEGFA expression in SCC compared to healthy skin samples, corresponding to previously reported findings [86]. Therefore, out of the assayed microRNAs, miR-361-5p seems most likely to contribute to elevated VEGFA mRNA levels in this type of cancer. It is well established that VEGFA, secreted by cultured keratinocytes, potently induces the proliferation of endothelial cells [87], implying that suppressors of VEGFA secretion may interfere with VEGFA-related functions in target cells. Moreover, previous findings in an experimental SCC model, demonstrating that elevated VEGFA levels were associated with increased tumor cell invasion and angiogenesis [10], indicate an indirect mechanism by which miR-361-5p might promote SCC progression. To gain first insights into possible functions of miR-361-5p, we analyzed the impact of altered miR-361-5p levels on proliferation (Figure S5A/B) and migration (Figure S5C/D) of A431 and HaCaT cells. However, the observed slight effects are likely not correlated with its targeting of VEGFA and may be the result of targeting other cancer related genes. Nevertheless, the presented data may provide a starting point for future studies investigating the potential roles of miR-361-5p on migration, proliferation and angiogenesis in relevant cell lines and SCC models. Next to a characterization of the miRNA’s functions *in vitro*, it may also be of interest – given the importance of VEGFA-related processes for wound healing and tissue regeneration – to study its function in an appropriate *in vivo* model (e.g. cutaneous wound healing).

With regard to the potential involvement of microRNA 361-5p in the etiology of SCC, an important question raised by our study is how the expression of the miRNA is regulated. One possibility is certainly that miR-361-5p transcription is dependent on its “host gene” *CHM*. However, while the levels of miRNA and ‘host gene’ expression correlated fairly well among the clinical samples, *CHM* and VEGFA levels did not. It is possible that additional regulation at the various steps of miRNA processing, as has been shown for a wide range of miRNAs [80], could lead to the observed discrepancies in the correlation of the assumed primary transcript (CHM) and the mature miRNA. Another possibility is that the miRNA bears its own promoter elements that could uncouple miRNA transcription from that of *CHM*. However, we are not aware of any literature on the transcriptional regulation of *CHM*. Therefore, detailed studies into the regulation of miRNA 361 should consider analyzing primary transcripts levels as well as those of miR-361-5p and -3p, their precursors, as well as CHM/Rab escort protein 1 protein levels. To our knowledge, CHM has not been implicated in either SCC or other skin-related diseases. However, there is some indication about a possible involvement of VEGFA in the etiology of choroideremia, a degenerative disease of the retina associated with the full or partial deletion of *CHM* and predominantly occurring in males: Vascular abnormalities in the endothelial cells of choroideremia patients have been previously reported [88]. A more recent study further identified VEGFA as one of a group of factors that is frequently expressed in a variable manner in the monocytes of choroideremia patients, and its levels correlated with disease severity [89]. Interestingly, many of the observed mutations/deletions in the *CHM* alleles of patients also affect miR-361-5p [90]. Loss-of-function mutations of miR-361-5p may thus potentially contribute to the observed variations in VEGFA levels. Finally, miRNA 361 is located on the × chromosome, which encodes 10% of all human miRNAs [91]. Although the significance of this enrichment is yet unknown, it has been proposed that the functions of X-linked miRNAs may explain some of the gender difference observed in immunity and related diseases, as well as cancers [92].

In conclusion, our findings further underline the presence of cancer-specific miRNA profiles [93]. Through microRNAome-wide expression profiling of cancers, it should therefore be possible to develop specific, miRNA-based clinical markers for diagnosis and the selection of treatment options for various types of cancers. With regards to SCC, studies with larger sample numbers and follow-up data should establish the value of miR-361-5p as a diagnostic and prognostic marker. As comprehensive data on the expression of miR-361-5p are currently unavailable, further studies should also reveal its association with other tumor types and targets, either acting in the same, or in different pathways.

## Materials and Methods

### Ethics Statement

The collection of specimens from clinically indicated excisions for this study was explicitly approved by the institutional review board (Kantonale Ethikkommission Zürich). Informed consent (both written and verbal) was obtained from patients for the use of their skin samples in this research project.

### Plasmids

A fragment comprising nucleotides 43,753,225 to 43,754,253 of human chromosome 6 (Build GRCh37/hg19, February 2009), containing nucleotides 926 to 1925 of the 3′-UTR of human VEGFA (isoform a, NM_001025366.2), was amplified from HeLa S3 genomic DNA using KOD Hot Start DNA Polymerase (Novagen, Cat. No. 71086-3) and the following primer pair (restriction sites are underlined): 5′-TCACTCGAGGTCCCGGCGAAGAGAAGAG-3′ (forward, containing *Xho*I site) and 5′-CATGCGGCCGCTCAATGGAGAAGGAGAAACCA (reverse, containing *Not*I site). The amplicon was cloned into pCR-Blunt II-TOPO (Invitrogen, Cat. No. K2800-20). The mutation in the putative miR-361-5p recognition element was introduced using the QuikChange I site-directed mutagenesis kit (Stratagene) according to the manufacturer’s recommendations. The following oligonucleotide and its reverse complement were used (the sequence complementary to the miRNA seed sequence is underlined): 5′-GTGTGTATATATATATATATATGTTTATGTATATATGTGATGATAAAATAGACATTGCTATTCTGTTTTTTATATGTAAAAACAAA-3′. After sequence verification, wild type and mutated VEGFA 3′-UTR fragments were subcloned into psiCHECK-2 (Promega) via *Xho*I and *Not*I restriction sites.

### Cell Culture and Tissue Samples

Primary human dermal blood vascular endothelial cells (BECs) and lymphatic endothelial cells (LECs) were isolated from neonatal foreskin as previously described [78], [79]. HEK293 [74], A431 [76] (both from American Type Culture Collection, Cat. Nos. CRL-1573 and CRL-1555, respectively), and HaCaT [77] (Cell Lines Service, Cat. No. 300493) cells were cultured in Dulbecco’s Modified Eagle Medium (DMEM; Invitrogen, Cat. No. 41966) supplemented with 10% fetal bovine serum (Invitrogen, Cat. No. 10270–106) and 1x antibiotic-antimycotic (Invitrogen, Cat. No. 15240–062). BECs and LECs were grown in endothelial basal medium (EBM; Lonza, Cat. No. CC-3121) supplemented with 20% fetal bovine serum, 1x antibiotic antimycotic, 2 mM L-glutamine (Invitrogen, Cat. No. 25030024), 10 µg/ml hydrocortisone (Sigma-Aldrich, Cat. No. H0396) and 25 µg/ml N-6,2′-O-dibutyryladenosine 3′,5′-cyclic monophosphate (Sigma-Aldrich, Cat. No. D0627) for up to 11 passages in plastic dishes coated with type I collagen (50 µg/ml in PBS; Advanced Biomatrix, Cat. No. 5005-B). All cells were grown in 5% CO_2_ at 37°C.

Squamous cell carcinoma (SCC) samples were obtained at the time of surgery. Normal skin was obtained from abdominoplastic reductive surgery. All specimens’ diagnoses were confirmed by a board-certified dermatohistopathologist. Four mm punch biopsies from SCC or normal skin were placed in preheated PBS at 60°C for 45 seconds, and then chilled on ice in 0.1% PBS for one minute, followed by mechanical separation of epidermis and dermis by scratching. The epidermis was homogenized in TRIzol reagent (Invitrogen, Cat. No. 15596–026) and stored at −80°C. RNA was extracted according to the manufacturer’s recommendations. Quantity and quality of extracted RNA was assessed by spectrophotometry with a NanoDrop 1000 (Thermo Fisher Scientific Inc.) and the 2100 Bioanalyzer (Agilent Technologies), respectively. All RNA samples had an RNA Integrity Number (RIN) of higher than 7.0.

### Luciferase Reporter Assays

20,000 HEK293 cells were reverse transfected with 20 ng of the indicated luciferase reporter constructs using polyethylenimine (Polysciences, Inc., Cat. No. 23966). For complex formation, DNA and polyethylenimine stock solution (1 mg/mL in water) were diluted to 20 µg/mL and 60 µg/mL, respectively, in Opti-MEM I (Invitrogen, Cat. No. 51985–026). Both solutions were incubated for 10 min at room temperature, and then mixed at equal volumes (mass ratio polyethylenimine to DNA = 3∶1; final polyethylenimine concentration = 30 µg/mL). After incubation for 20 min at room temperature, solutions were diluted 1∶10 in DMEM (Invitrogen, Cat. No. 41966) to achieve a final DNA concentration of 1 µg/mL. 20 µL of the transfection mixes were added to each well of a 96-well plate, followed by the addition of 80 µL of cell suspension in DMEM (2.5×10^5^ cells/mL). 16 hours after transfection of the luciferase reporter plasmid, the cells were further transfected with 50 nM of Pre/Anti-miR-361-5p or Pre−/Anti-miR Negative Control #1 (Applied Biosystems, Table S4) by using siPORT NeoFX (Applied Biosystems) according to the manufacturer’s recommendations. All transfections were performed in triplicate. After 48 hours, the medium was aspirated and cells were lysed with a mixture of 15 µL Luciferase Assay Reagent II (Promega) and 15 µL nuclease-free water (Invitrogen, Cat. No. 10977). Firefly luciferase activity was measured after 10 min. Then, 15 µL Stop & Glo Reagent (Promega) were added and *Renilla* luciferase activity was measured after 10 min. Luciferase activity measurements were performed in an LMAX II 384 luminometer (Molecular Devices), with 5 seconds integration time. For each triplicate, the mean *Renilla*/firefly ratio was calculated.

### ELISA

20,000 A431 or HaCaT cells were reverse transfected with 10, 30, 50 or 100 nM Pre−/Anti-miR-361-5p or Pre−/Anti-miR Negative Control #1 (Applied Biosystems, Table S4) by using siPORT NeoFX (Applied Biosystems) according to the manufacturer’s recommendations. Transfections were performed in triplicate in 96-well plates. Where indicated, TNF-α (Peprotech, Cat. No. 300–01A) was added at 10 ng/mL immediately after transfection to induce VEGFA expression. 24 hours after transfection, supernatants were collected and centrifuged to remove cell debris (1000 g for 3 min at room temperature). VEGFA protein levels were determined using the Human VEGF-A Platinum ELISA kit (eBioscience, Cat. No. BMS277) according to the manufacturer’s recommendations. After subtraction of blank values, triplicates were averaged and quantified using a standard curve prepared from serial dilutions of purified VEGFA. Data were normalized by cell number.

### Flow Cytometry

125,000 A431, HaCaT or HEK293 cells were reverse transfected with 10, 30 or 100 nM Cy3 dye-labeled Pre- or Anti-miR Negative Control #1 (Applied Biosystems, Table S4), or mock-transfected, using siPORT NeoFX (Applied Biosystems) according to the manufacturer’s recommendations. Transfections were performed in triplicate in 24-well plates. After 24 hours, cells were de-attached with 0.05% trypsin-EDTA (Invitrogen, Cat. No. 25300-054) and washed with PBS. Dye-labeled molecules were excited with a blue laser (excitation wavelength = 488 nm) and analyzed with a FACScan flow cytometer (Beckon Dickinson). At least 5000 events were recorded for each sample. Data were analyzed with WinMDI 2.8.

### Quantitative Reverse Transcription PCR

For each reaction, cDNA was prepared from 10 ng total RNA using the TaqMan MicroRNA Reverse Transcription Kit (Applied Biosystems) for miRNA detection, or the High Capacity cDNA Reverse Transcription Kit (Applied Biosystems) for mRNA detection. MicroRNA and gene expression assays were purchased from Applied Biosystems (Table S5). Quantitative PCR reactions were performed in quadruplicates, using FastStart TaqMan Probe Master (Rox; Roche) in an AB 7900 HT Fast Real-Time PCR System (Applied Biosystems). Quantification was performed using the 2^−ΔΔCT^ method [94], with RNU6B and ACTB as references for the normalization of miRNA and mRNA expression levels, respectively. Based on the manufacturer’s assertions, equal amplification efficiencies of close to 100% were assumed for all assays, thus justifying the application of the 2^−ΔΔCT^ analysis method and the comparisons between different microRNA and gene expression assays.

### Monolayer Wound Healing and Proliferation Assay

For proliferation assays, 5,000 A431 or HaCaT cells were reverse transfected with 50 nM Pre−/Anti-miR-361-5p or Pre-/Anti-miR Negative Control #1 (Applied Biosystems, Table S4) by using siPORT NeoFX (Applied Biosystems) according to the manufacturer’s recommendations. Transfections were performed in eight replicates per condition in black, clear-bottom 96-well plates (Corning, Cat. No. CLS3603). Cells were incubated in DMEM supplemented with 1% fetal bovine serum. 72 hours after transfection, cells were incubated with 4-methylumbelliferyl heptanoate (Sigma-Aldrich, Cat. No. M2514) as previously described [95]. Fluorescence intensities were measured with a SpectraMax Gemini EM microplate reader (Molecular Devices).

For monolayer wound healing assays, 125,000 A431 or HaCaT cells were reverse transfected with 50 nM Pre-/Anti-miR-361-5p or Pre-/Anti-miR Negative Control #1 (Applied Biosystems, Table S4) by using siPORT NeoFX (Applied Biosystems) according to the manufacturer’s recommendations. Transfections were performed in triplicate in 24-well plates. Monolayer wound healing assays were performed as previously described [96]. Briefly, 14 hours after transfection, the medium was replaced with DMEM supplemented with 1% fetal bovine serum, and cells were incubated for 12 hours. Cell monolayers were scratched crosswise with a sterile 200 µL pipette tip and imaged at 5× magnification using an Axiovert 200 M microscope with an AxioCam MRm camera (Carl Zeiss AG) immediately after scratching, as well as after 18 hours of incubation. Open wound areas were quantified using the TScratch software [96].

### MicroRNA Target Gene Prediction and Pathway Analysis

Predictions of human miRNA targets were downloaded from microRNA.org [54] (August 2010 release, http://www.microrna.org/, accessed: August 12^th^, 2011; all predictions with mirSVR of less than -0.1 were considered), TargetScan [55] (Release 5.2; http://www.targetscan.org/, accessed: August 12^th^, 2011; all predicted miRNA recognition elements were considered, regardless of conservation), DIANA-microT v3.0 [56] (Release 3.0, http://diana.cslab.ece.ntua.gr/microT/, accessed: August 12^th^, 2011), miRDB [57] (Release 3.0, http://mirdb.org/miRDB/, accessed: August 12^th^, 2011), and MicroCosm [58] (Release 5, www.ebi.ac.uk/enright-srv/microcosm/, accessed: August 12^th^, 2011). The miR-361-5p sequence was obtained from miRBase [97] (Release 17, http://www.mirbase.org/, accessed: May 5^th^, 2011). RNAhybrid [59] (http://bibiserv.techfak.uni-bielefeld.de/rnahybrid/) was used online, with default settings and the following sequences: UUAUCAGAAUCUCCAGGGGUAC (miR-361-5p, microRNA) and UGUAUAUAUGTGAUUCUGAUAAA (VEGFA 3′-UTR fragment containing the putative miR-361-5p MRE, target RNA). For the pathway analysis, predicted targets for miR-361-5p were converted to Entrez identifiers using DAVID [98] (http://david.abcc.ncifcrf.gov/), if not present in the respective outputs. Results were pooled and filtered for unique records, and subjected to gene set enrichment analysis with PANTHER [99] (http://www.pantherdb.org/tools/compareToRefListForm.jsp). For VEGFA pathway analysis, the KEGG PATHWAY database [100] (http://www.genome.jp/kegg/pathway.html) was consulted. All services were used with default settings.

## Supporting Information

Figure S1 **Overview of the human VEGFA 3′-UTR, hsa-mir-361 and their predicted hybrid structure.** (A) UCSC genome browser [101] view of the genomic locus encoding the human VEGFA 3′-UTR. Indicated are the terminal exon of the VEGFA gene, PhyloP and PhastCons conservation scores, GC content, the 3′-UTR fragment common to all isoforms, and the 3′-UTR region subcloned downstream of the luciferase reporter used in this study. Moreover, the relative locations of the recognition elements for miRNAs demonstrated to be able to regulate either human or mouse VEGFA (see introduction for details) are highlighted. The miR-361-5p binding site analyzed in this study is indicated in red. Please note that predicted MREs whose activities have not been unambiguously identified by mutational analysis are listed separately. (B) Secondary structure of the hybrid between miR-361-5p and the putative miRNA recognition element within the VEGFA 3′-UTR, as predicted by RNAhybrid [59]. The calculated free energy of the interaction is indicated.
(TIF)

Figure S2 **Pathway analysis of predicted miR-361-5p targets.** Target predictions were obtained from the following web services: microRNA.org [54], TargetScan [55], DIANA-microT [56], miRDB [57], and MicroCosm [58]. Results were pooled and converted to uniform gene identifiers using the DAVID web service [98]. A manually curated representation of the VEGF signaling pathway available at KEGG [100] was color-coded according to the number of algorithms that predict an individual gene to be targeted by miR-361-5p.
(TIF)

Figure S3 **MicroRNA mimics and antisense inhibitors are readily taken up by HEK293, A431 and HaCaT cells.** Cells were transfected with either 0 (mock), 10, 30 or 100 nM of Cy3-labeled Pre-miR or Anti-miR oligonucleotides and analyzed by flow cytometry. Experiments were performed in triplicate. (A) For each cell line, dot plots of mock-transfected cells (left panel) indicate the populations subjected to fluorescence analysis. The fluorescence distributions of gated cells are plotted for a single replicate, both for Pre-miR (middle) and Anti-miR-transfected cells (right). The fluorescence thresholds for positive cells are indicated (M1). The efficiencies of transfection are represented as the mean fractions of fluorescent cells (“M1 positive cells”) (B) and the mean geometric means (C) within the gated populations ± S.D.
(TIF)

Figure S4 **Relative changes in expression of selected mRNAs and mature miRNAs in healthy skin and SCCs.** For each group, five samples were analyzed by qRT-PCR, representing ten individuals. Experiments were performed in quadruplicates. Data are based on C_T_ values normalized to ACTB and RNU6B for mRNAs and miRNAs, respectively. (A) For each of the indicated assays, the fold difference in expression with regard to the reference is plotted for each of the healthy skin samples. Horizontal bars represent means of all healthy skin samples. (B) For each assay and for each of the cutaneous squamous cell carcinoma samples the fold difference in expression to the average of the healthy skin samples is indicated. Horizontal bars represent means of all squamous cell carcinoma samples.
(TIF)

Figure S5 **Effects of altered miR-361-5p levels on the proliferative and migratory properties of skin-derived cells.** The effects of overexpression and inhibition of miR-361-5p on proliferation and migration of A431 and HaCaT cells were assessed with (A) 4-methylumbelliferone-based proliferation and (B) monolayer wound healing (“scratch”) assays. Cells were transfected with 50 nM of miR-361-5p mimics, antisense inhibitors, or controls, and made responsive by exposition to reduced serum concentrations (72 and 12 hours, respectively). (A) 72 hours after transfection, relative fluorescence intensities were measured in eight replicates after incubating cells with 4-methylumbelliferyl heptanoate. (B) Cell monolayers were scratched and imaged after 0 and 18 hours. Relative differences in open wound areas (in %) were automatically quantified using TScratch [96]. For all assays, three independent experiments were performed. Mean values ± S.D. from a representative experiment are plotted. Two-tailed, unpaired *t*-tests were used to calculate P values (one and two asterisks denote P values<0.05 and<0.01, respectively).
(TIF)

Table S1 **MicroRNAs predicted to target VEGFA.** A comprehensive list indicating the miRNAs predicted to have recognition elements in the VEGFA 3′-UTR by any of the algorithms used in this study are indicated in the first sheet. For each MRE, the genomic coordinates, the 3′-UTR region as defined in the main text, the relative position to the start site of the 3′-UTR, the particular algorithms and the total number of algorithms predicting the MRE, as well as the total number of algorithms for which target predictions for the corresponding miRNA were available in the accessed information, are indicated. For MREs previously subjected to experimental analysis, the corresponding references are indicated. An analysis of the density of predicted MREs in the whole 3′-UTR as well as the defined 3′-UTR regions is given in the second sheet.
(XLSX)

Table S2 **Predicted miR-361-5p targets.** Target predictions were obtained from the following web services: microRNA.org [54], TargetScan [55], DIANA-microT [56], miRDB [57], and MicroCosm [58]. Results were pooled and converted to uniform gene identifiers using the DAVID web service [98]. For each gene, the particular algorithms and the total number of algorithms predicting it are indicated. Genes acting in the VEGF pathway according to KEGG [100] are shown.
(XLSX)

Table S3 **Gene set enrichment analysis of predicted miR-361-5p targets.** Target predictions were obtained from the following web services: microRNA.org [54], TargetScan [55], DIANA-microT [56], miRDB [57], and MicroCosm [58]. Results were pooled and converted to uniform gene identifiers using the DAVID web service [98]. Putative targets were compared to a human reference gene list and analyzed for pathway enrichment using PANTHER [99]. Pathways significantly enriched among predicted miR-361-5p targets are given. For each pathway, the number of genes from the reference list, the number of genes from the putative target list, the number of genes expected by chance, and the P value are indicated. P values are not Bonferroni-corrected. The VEGF pathway is highlighted.
(XLSX)

Table S4 **Overview of microRNA mimics and antisense inhibitors used in this study.** Ordering information and, where applicable, identifiers and sequences of the mature miRNAs are indicated.
(XLSX)

Table S5 **Overview of quantitative reverse transcription PCR assays used in this study.** In addition to the ordering information, identifiers of the targeted genes and assayed transcripts are given. Where available, the binding regions of the primers and probes as well as the amplicon lengths are indicated.
(XLSX)
