# Supplementary figures and images for: The Expression Levels of MicroRNA-361-5p and Its Target VEGFA Are Inversely Correlated in Human Cutaneous Squamous Cell Carcinoma
Source: PLoS One. 2012 Nov 14;7(11):e49568. doi: 10.1371/journal.pone.0049568 (PMC3498195; doi:10.1371/journal.pone.0049568)

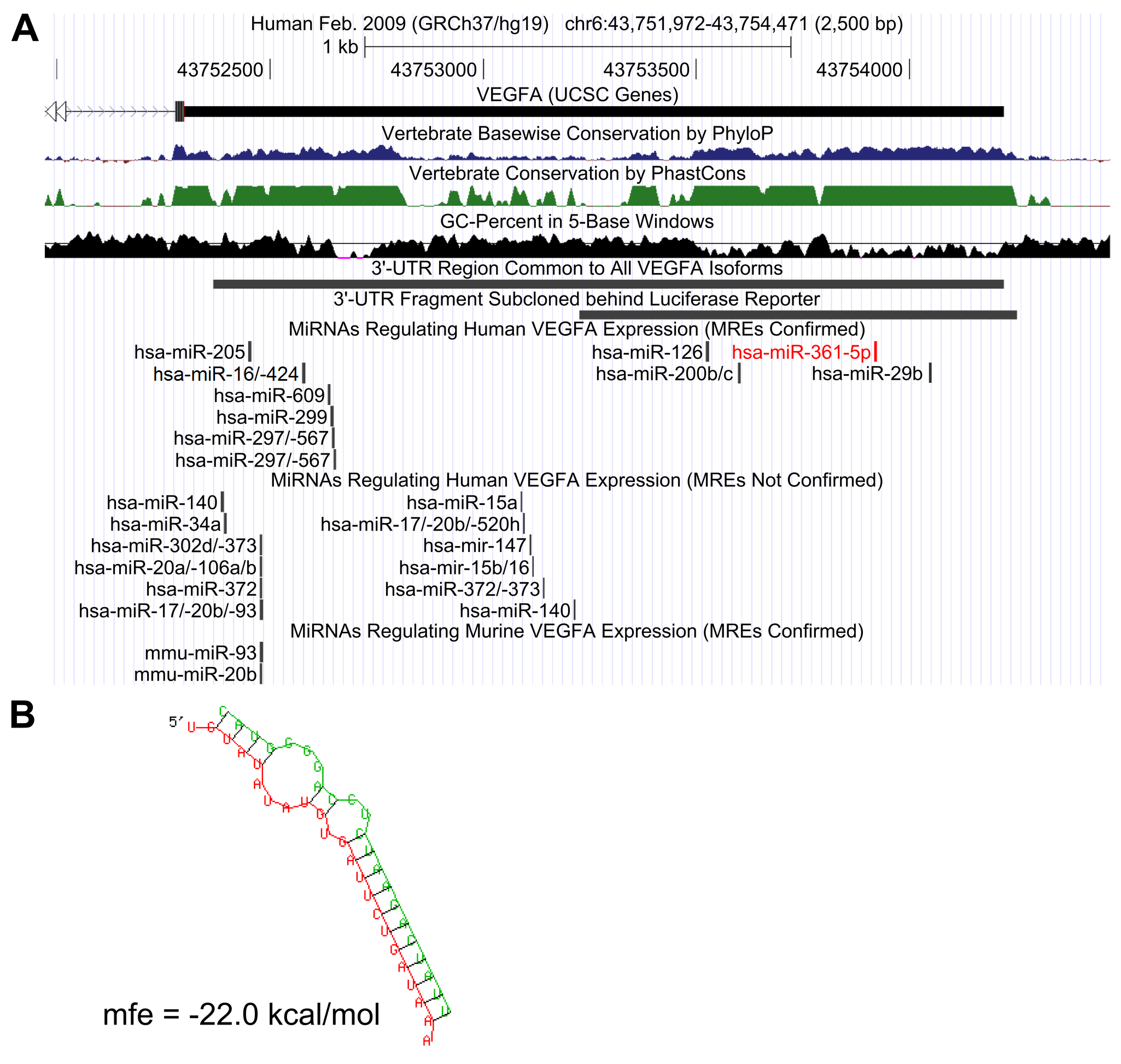

Supplement: Figure S1 — Overview of the human VEGFA 3′-UTR, hsa-mir-361 and their predicted hybrid structure. (A) UCSC genome browser [101] view of the genomic locus encoding the human VEGFA 3′-UTR. Indicated are the terminal exon of the VEGFA gene, PhyloP and PhastCons conservation scores, GC content, the 3′-UTR fragment common to all isoforms, and the 3′-UTR region subcloned downstream of the luciferase reporter used in this study. Moreover, the relative locations of the recognition elements for miRNAs demonstrated to be able to regulate either human or mouse VEGFA (see introduction for details) are highlighted. The miR-361-5p binding site analyzed in this study is indicated in red. Please note that predicted MREs whose activities have not been unambiguously identified by mutational analysis are listed separately. (B) Secondary structure of the hybrid between miR-361-5p and the putative miRNA recognition element within the VEGFA 3′-UTR, as predicted by RNAhybrid [59]. The calculated free energy of the interaction is indicated. (TIF) [file pone.0049568.s001.tif]

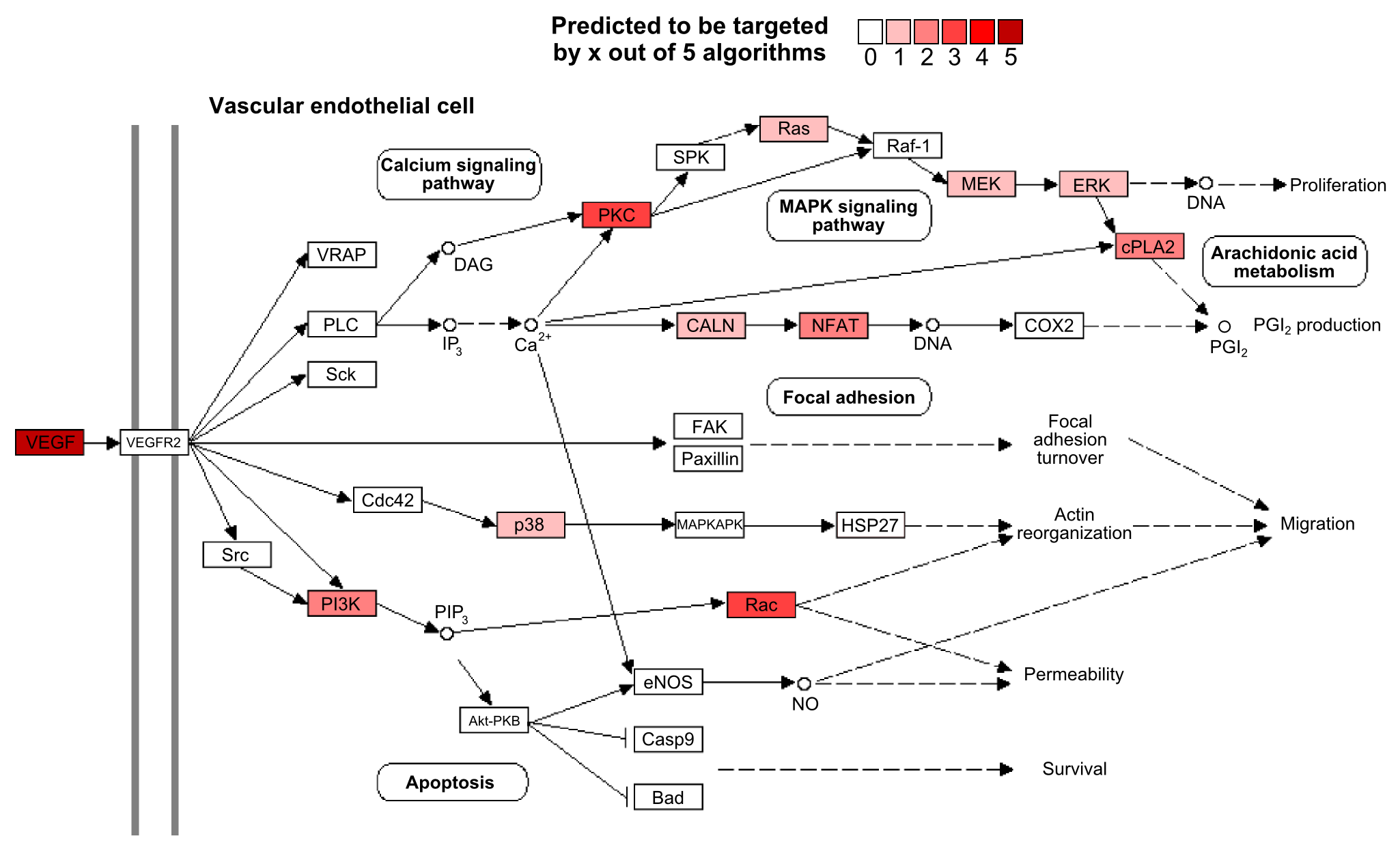

Supplement: Figure S2 — Pathway analysis of predicted miR-361-5p targets. Target predictions were obtained from the following web services: microRNA.org [54], TargetScan [55], DIANA-microT [56], miRDB [57], and MicroCosm [58]. Results were pooled and converted to uniform gene identifiers using the DAVID web service [98]. A manually curated representation of the VEGF signaling pathway available at KEGG [100] was color-coded according to the number of algorithms that predict an individual gene to be targeted by miR-361-5p. (TIF) [file pone.0049568.s002.tif]

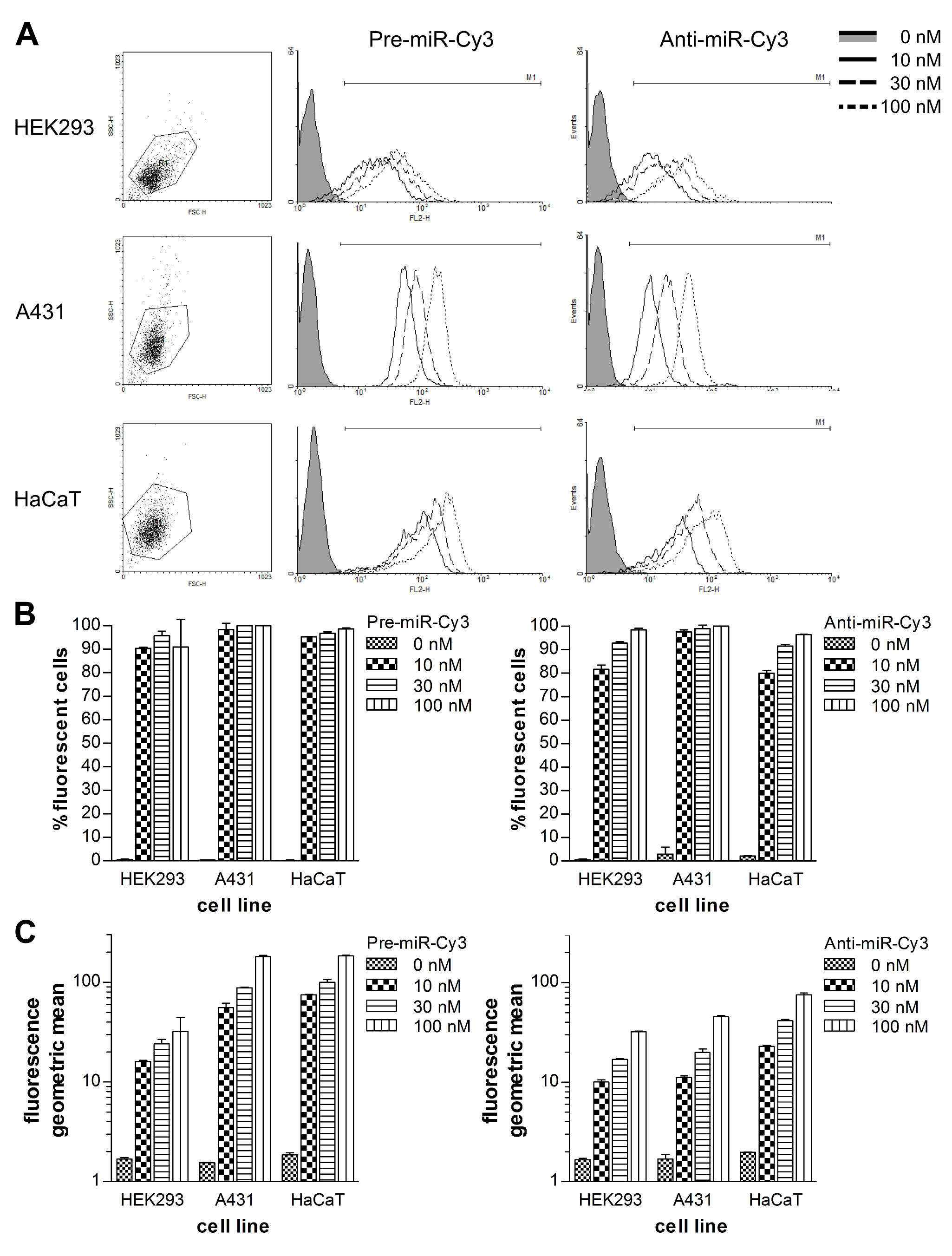

Supplement: Figure S3 — MicroRNA mimics and antisense inhibitors are readily taken up by HEK293, A431 and HaCaT cells. Cells were transfected with either 0 (mock), 10, 30 or 100 nM of Cy3-labeled Pre-miR or Anti-miR oligonucleotides and analyzed by flow cytometry. Experiments were performed in triplicate. (A) For each cell line, dot plots of mock-transfected cells (left panel) indicate the populations subjected to fluorescence analysis. The fluorescence distributions of gated cells are plotted for a single replicate, both for Pre-miR (middle) and Anti-miR-transfected cells (right). The fluorescence thresholds for positive cells are indicated (M1). The efficiencies of transfection are represented as the mean fractions of fluorescent cells (“M1 positive cells”) (B) and the mean geometric means (C) within the gated populations ± S.D. (TIF) [file pone.0049568.s003.tif]

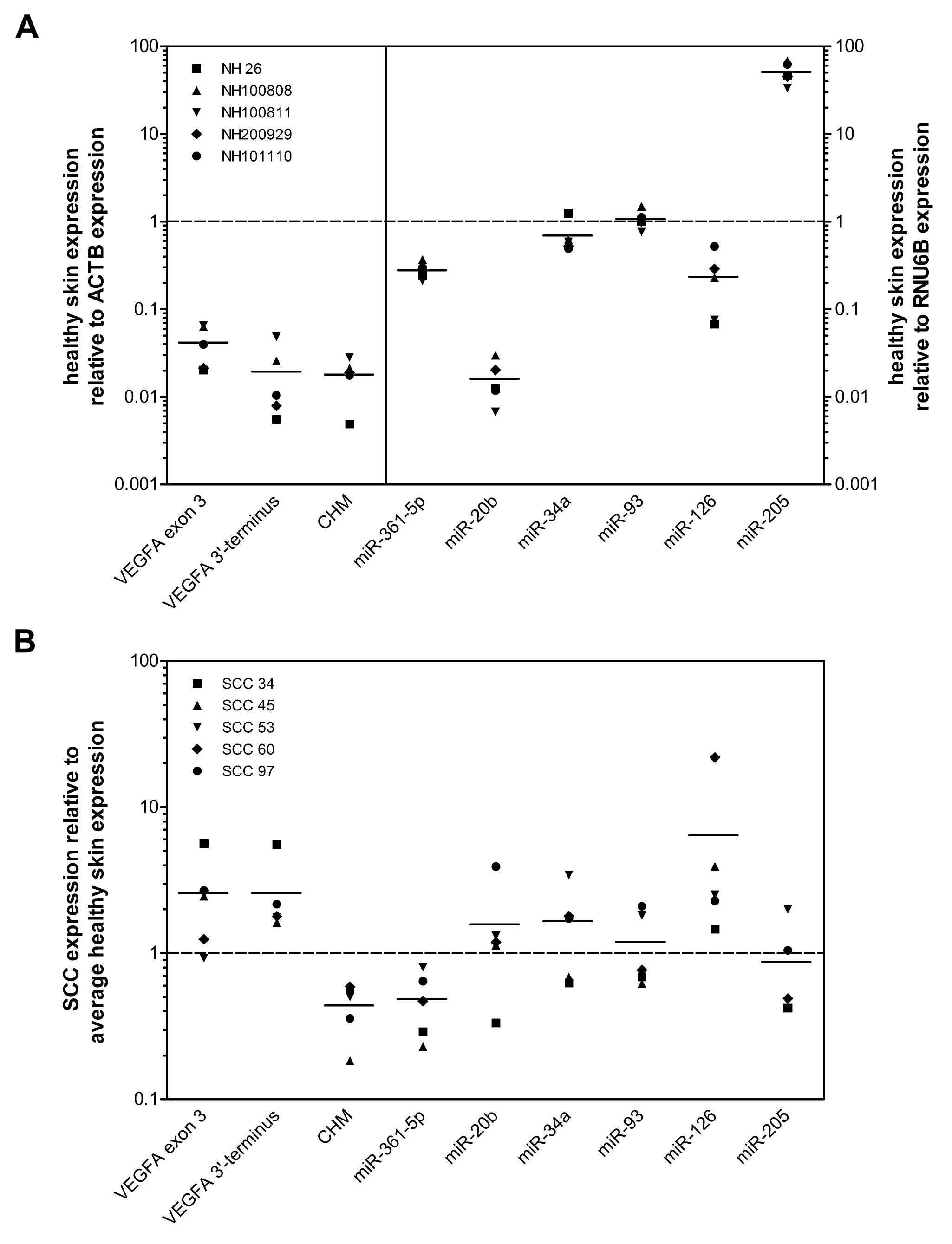

Supplement: Figure S4 — Relative changes in expression of selected mRNAs and mature miRNAs in healthy skin and SCCs. For each group, five samples were analyzed by qRT-PCR, representing ten individuals. Experiments were performed in quadruplicates. Data are based on CT values normalized to ACTB and RNU6B for mRNAs and miRNAs, respectively. (A) For each of the indicated assays, the fold difference in expression with regard to the reference is plotted for each of the healthy skin samples. Horizontal bars represent means of all healthy skin samples. (B) For each assay and for each of the cutaneous squamous cell carcinoma samples the fold difference in expression to the average of the healthy skin samples is indicated. Horizontal bars represent means of all squamous cell carcinoma samples. (TIF) [file pone.0049568.s004.tif]

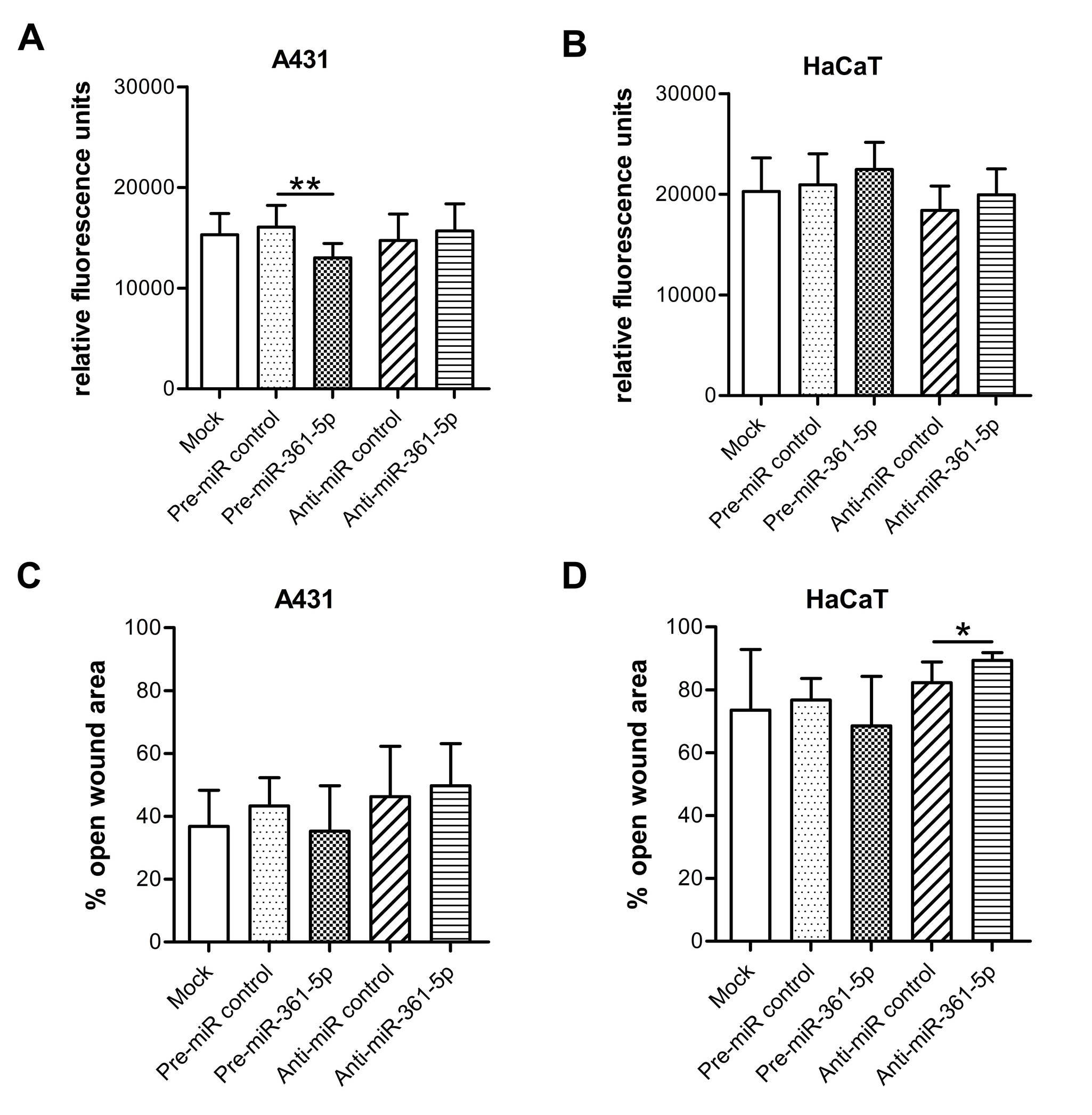

Supplement: Figure S5 — Effects of altered miR-361-5p levels on the proliferative and migratory properties of skin-derived cells. The effects of overexpression and inhibition of miR-361-5p on proliferation and migration of A431 and HaCaT cells were assessed with (A) 4-methylumbelliferone-based proliferation and (B) monolayer wound healing (“scratch”) assays. Cells were transfected with 50 nM of miR-361-5p mimics, antisense inhibitors, or controls, and made responsive by exposition to reduced serum concentrations (72 and 12 hours, respectively). (A) 72 hours after transfection, relative fluorescence intensities were measured in eight replicates after incubating cells with 4-methylumbelliferyl heptanoate. (B) Cell monolayers were scratched and imaged after 0 and 18 hours. Relative differences in open wound areas (in %) were automatically quantified using TScratch [96]. For all assays, three independent experiments were performed. Mean values ± S.D. from a representative experiment are plotted. Two-tailed, unpaired t-tests were used to calculate P values (one and two asterisks denote P values<0.05 and<0.01, respectively). (TIF) [file pone.0049568.s005.tif]
